# Effects of *L. reuteri* NBF 2 DSM 32264 Consumption on the Body Weight, Body Condition Score, Fecal Parameters, and Intestinal Microbiota of Healthy Persian Cats

**DOI:** 10.3390/vetsci11020061

**Published:** 2024-02-01

**Authors:** Benedetta Belà, Daniele Di Simone, Giulia Pignataro, Isa Fusaro, Alessandro Gramenzi

**Affiliations:** 1 Department of Veterinary Medicine, University of Teramo, Piano d’Accio, 64100 Teramo, Italy; gpignataro@unite.it (G.P.); ifusaro@unite.it (I.F.); agramenzi@unite.it (A.G.); 2 Department of Economics and Finance, University of Bari, Largo Abbazia Santa Scolastica 53, 70124 Bari, Italy; daniele.disimone93@gmail.com

**Keywords:** probiotic, fecal quality, intestinal microbiota, Persian cats

## Abstract

**Simple Summary:**

The intestinal microbiota, i.e., the set of microorganisms that colonize the gastrointestinal tract, are fundamental for animal health. To ensure the correct functioning of the organism, it is essential that the pathogenic and beneficial microorganisms that make up the intestinal microbiota are in balance with each other; in fact, an alteration in the microbiota composition, called dysbiosis, is associated with several pathologies. Specific bacterial strains with probiotic action are used to preserve and maintain the intestinal microbiota in a balanced condition (eubiosis), preventing the dysbiosis onset. To date, no study has analyzed the effects of the probiotic *Lactobacillus reuteri* on the health of cats. The authors of the present study have observed how the administration of *L. reuteri* DSM 32264 in Persian cats manages to promote intestinal well-being by decreasing pathogenic bacteria such as *Escherichia coli* and increasing Lactobacilli. Furthermore, the administration of *L. reuteri* did not cause changes in the body weight of cats, promoting the formation of compact and well-formed stools indicative of a balanced intestinal ecosystem. The results of this study show how *L. reuteri* DSM 32264 can be a valid aid in maintaining correct intestinal balance and well-being in cats.

**Abstract:**

In the literature, there are several studies showing the effects of different probiotic administrations in dogs, while there is limited information about their effects in cats. Furthermore, there are no studies that examined the effects of the probiotic strain *Lactobacillus reuteri* on cats’ welfare, especially considering a specific breed. In this study, the effects of *L. reuteri* NBF 2 DSM 32264 on body weight, body condition score (BCS), and fecal parameters (fecal score and fecal moisture) of healthy Persian cats were assessed; additionally, a microbiological analysis was carried out to quantify bacterial species like *Escherichia coli* (for the total coliform count) and Lactobacilli. The administration of *L. reuteri* NBF 2 DSM 32264 showed no alteration in the body weight and body condition score of Persian cats. The fecal moisture decreased at the end of the study and the values of fecal score were improved. Moreover, at the end of the study period, an increase in *Lactobacilli* (*p* > 0.001) was observed. The data collected report the ability of *L. reuteri* NBF 2 DSM 32264 to improve fecal quality parameters in healthy adult Persian cats, leading to an increase in *Lactobacilli* and a reduction in total coliforms.

## 1. Introduction

In recent years, the gut microbiota has attracted great interest due to the important role it plays in health and disease; in fact, several studies in humans and animals have highlighted how its alterations can be associated with various pathologies [1,2,3,4,5]. Although specific pathogenic bacteria are well known in dogs and cats, many of them are sometimes found in the same frequencies, even in healthy subjects; their cause–effect relationships remain to be better clarified [6]. Recent studies showed how changes in the composition of the intestinal microbiota can cause acute and/or chronic gastrointestinal disorders in dogs and cats, underlining how the intestinal microbiota is mainly involved in the pathogenesis of chronic enteropathies such as inflammatory bowel disease (IBD). This pathology is very frequent in dogs and cats, and, as in humans, the exact cause of canine and/or feline IBD is still unknown. The response to a specific treatment can be used to classify the enteropathy into food-responsive diarrhea, antibiotic-responsive diarrhea, and steroid-responsive diarrhea; the first one often can be treated with a specific diet combined with the administration of specific beneficial bacteria with probiotic action [7,8], facilitating the re-balance of the intestinal microbiota altered during these pathologies. Human chronic enteropathies usually cause a decrease in the bacterial phyla Firmicutes and Bacteroidetes with an increase in Proteobacteria and Actinobacteria; similar changes were also observed in dogs, but regarding cats, though not many studies have yet been conducted, an increase in Proteobacteria accompanied by a decrease in *Bifidobacterium* and *Bacteroides* spp. was observed [9]. Furthermore, a possible susceptibility of the innate immune system was discovered in dogs with chronic intestinal inflammation, showing an altered expression of Toll-like receptors (TLRs) 2 and 4 [10] and a single nucleotide polymorphism that leads to an over-responsiveness of TLR-5 to flagellin, as seen in German Shepherd dogs [11]. Additionally, a lower expression of CD 11c (+) cells was found in dogs with IBD. There are also specific dog breeds that are more predisposed to chronic intestinal inflammation, such as Rottweiler, Border Collie, Boxer and Weimaraner [12]; unfortunately for cats, we do not have such detailed information, nor do we know which breeds are most likely to develop the pathology. The present study considered a specific breed of cat, the Persian one, analyzing the effects a probiotic strain can have on it. The probiotic strain under study was *Lactobacillus reuteri* NBF2 DSM 32264. The beneficial properties of *L. reuteri* at the intestinal microbiota level are already well known [13,14,15,16]; however, as already mentioned, there are no studies on cats, and this experiment aims to consider how this specific bacterial strain acts on the fecal and microbiological parameters of cats. To be considered a probiotic, a *bacterium* must arrive alive at the intestinal level, colonizing the mucosa and competing with pathogenic bacteria for nutrients. Furthermore, through the release of metabolites such as short-chain fatty acids (SCFAs), it causes a lowering of intestinal pH, creating an environment that is even more hostile to pathogens as well as providing volatile fatty acids, which are very important for the health of colonocytes such as butyrate. These actions, in addition to the production of vitamins and the metabolization of bile acids, maintain a balanced intestinal ecosystem, which is fundamental for preventing dysbiosis and other alterations that cause pathologies. As previously mentioned, probiotics are often administered in cases of food-responsive enteropathy, thanks to their ability to restore the balance of intestinal bacterial species, thereby improving fecal parameters such as fecal score (FS) and fecal moisture (FM) by promoting the excretion of well-formed and compact stools, a symptom of a healthy intestinal environment. This study aims to analyze if *L. reuteri* NBF 2 DSM 32264 can exert the aforementioned effects on Persian cats by examining its effects at the intestinal microbiota level through the count of total lactobacilli and coliforms to see if *L. reuteri* NBF 2 DSM 32264 can promote a correct intestinal balance and can be eventually used in specific diets to counteract dysbiosis and related inflammatory pathologies.

## 2. Material and Methods

### 2.1. Ethical Statement

The present study was performed following what is reported in the directive 2010/63/EU, article 1 (paragraph 5f), and did not cause any health risk or suffering to the animals as it involves the administration of a natural substance. All cats enrolled in this study were owned and subjected to regular veterinary checks.

### 2.2. Animals and Study Design

In total, 12 healthy Persian cats, 4 males and 8 non-pregnant females, age > 1 year, were involved in this study and randomly assigned to the control group (CTR; *n* = 6; male: female = 1:1) and experimental group (LACTO; *n* = 6; male: female = 1:5); the trial lasted a total of 49 days: 35 days of study and two weeks of acclimatization.

To minimize the selection error bias, cats were divided randomly (SurveyMonkey Excel) into two groups: the control group and the experimental group. Group assignment was organized according to kennel management standard procedure. The experimental group was supplemented with *L. reuteri* NBF 2 DSM 32264, while the control group did not receive the probiotic and was supplemented with maltodextrin used as a placebo. Table 1 reports the age, sex and body weight of each cat belonging to the two groups. Cleaning and disinfecting procedures of the single fences were carried out, and the animals were individually tabulated. Moreover, the animals were evaluated daily by a veterinarian for any health and welfare concerns throughout the experimental period.

### 2.3. Feed Supplement and Diet

Both groups of cats had free access to potable water and were fed with a commercial pet food (Farmina N&D prime feline chicken & pomegranate adult) once a day, based on their maintenance energy requirements (adult cats: 100 kcal × BW^0.67^ kg) [17] during the entire experimental period. Table 2 reports the pet food analytical components. Cats belonging to the LACTO group received commercial feed with the addition of 10 g/100 kg of *L. reuteri* NBF 2 DSM 32264, corresponding to 5 × 10^9^ colony-forming units (CFUs)/kg of food, and the consumption for each cat was measured by weighing the residue before the next day’s meal was administered. The process to obtain the right probiotic amount consisted of using 50 g of the feed additive (standard concentration ≥ 1.0 × 10^11^ CFU/g) pre-mixed in the laboratory with 9950 g of maltodextrins. Then, a total of 20 g of this pre-mixture was added daily to each 980 g of commercial feed in the bowl. The CTR group received the commercial diet, with the addition of 20 g of maltodextrin (placebo) in 980 g of dog feed. Five samples of the feed belonging to the CTR and LACTO groups were sent to the laboratory to verify the number of *Lactobacilli* in the preparation and the absence of undesired bacteria.

### 2.4. Data Collection

Bodyweight (BW) and body condition score (BCS) were recorded at the beginning of the study (day 0, T0), after one week (T1), after two weeks (T2), after three weeks (T3), after four weeks (T4) and at the end of the experiment (T5), according to the American Animal Hospital Association (AAHA) Nutritional Assessment Guidelines for Dogs and Cats [18]. The BW of each animal was measured by the same person at the same time (the morning before feed administration) with the same instrument. At the same time, a BCS assessment was carried out by visual examination and palpation of the animal on a scale between 1 and 9, where a score of 4 or 5 reflects the ideal body condition depending on the breed [19]. To evaluate the effect of the probiotic on fecal quality, an assessment of fecal score (FS) and fecal moisture (FM) was performed; the fecal score was evaluated using a 7-point scoring chart according to the Nestle Purina Fecal Scoring System at all six sampling times (T0–T5), together with the fecal moisture. Furthermore, some GI bacterial species were identified, and their species count was investigated. Fecal samples were collected at T0, T1, T2, T3, T4 and T5 and then stored at +4 °C until they were brought to the laboratory, where they were stored at −20 °C. Then, 5–10 g of stool was weighed and dried in an oven at a temperature of 105 °C–110 °C for 20–24 h, cooled down in a desiccator for another 20–24 h, after which the fecal moisture content was calculated as lost weight after desiccation. One gram of fresh stool was used to carry out the microbiological analysis performed at T0, T1, T3 and T5. Briefly, fresh stool was diluted in a sterile saline solution with a ratio of 1:10 and then vortexed for two minutes to create a homogeneous suspension, which was then plated onto different culture media for total bacterial counts and identification. Specifically, for *Escherichia coli* and total coliforms, the eosin methylene blue agar was used (Oxoid, Milan, Italy); after 24 h of incubation at 37 °C, *E. coli* colonies showed growth with a green metallic reflex, while coliforms showed growth with blue, red, or uncolored colonies. For lactobacilli identification, the Man Rogosa and Sharpe agar (Oxoid, Milan, Italy) was used, and the plates were incubated for 48 h at 37 °C under anaerobic conditions. All the analysis was performed in duplicate.

### 2.5. Statistical Analysis

For the statistical analysis, a mixed model with repeated measurements has been used, which allows the estimation of the parameters considering both random effect and fixed effect [20]. The model has been estimated as the following:

yi,j,k = μ + Si + Gj + Tk + Gj × Tk + ei,j,k

where y = dependent variable (FM, FS, BW, BCS, LB, COLI); μ = overall mean; Si = fixed effect of the ith sex (i = 1, 2); Gj = fixed effect of the jth group (j = 1, 2); Tk = fixed effect of the kth time (k = 0, 5); and ei,j,k = error. The software used was R Core Team 4.0.0 (2020), R: A language and environment for statistical computing [21]. For the different analyses, the mixed model [22] and the least squares [23] were used. Time was used as a repeated measurement, and therefore each subject was analyzed in every different temporal instant. The autoregressive covariance structure was used. Least squares means were estimated and statistically tested using Student’s *t*-test (with Tukey *p*-value adjustment). To be able to describe the goodness of the fit of the mixed model, we used the R squared described by Nakagawa et al. [24]. No outliers or missing data were found.

## 3. Results

**Figure 1:**
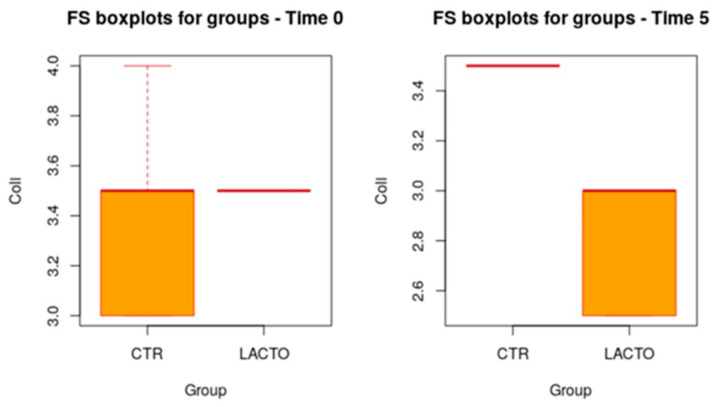
Box plot showing the effect of *Lactobacillus reuteri* NBF 2 DSM 32264 addition to the diet on the fecal score (FS) of Persian cats during the overall period (*p* = 0.0068; *t*-test). CTR, control group; LACTO, experimental group.

All cats were in good health condition during the trial, and no side effects or cases of death were recorded; furthermore, no residual pet food was found after consumption throughout the experimental period. BW and BCS values did not change during the study in both groups of cats (Table 3A,B) and the animals maintained an ideal body condition with a score between 4.5 and 5. In the last two weeks of the trial (T4 and T5), a lower humidity content was found in the fecal sample of cats treated with *L. reuteri* NBF 2 DSM 32264, compared with the value recorded in the control group (*p* = 0.001; Table 4); the beneficial effect of *Lactobacillus reuteri* NBF 2 DSM 32264 was also confirmed by the fecal score (FS) values (Table 5; Figure 1). The control group showed higher FS values than the group of cats treated with the probiotic, which showed much lower values: 3.53 ± 0.43 at the beginning of the trial and 2.86 ± 0.43 at the end of the experiment. The decrease of about 0.7 points on a scale of 1 to 7 can certainly have implications for the intestinal health of cats with an important biological relevance [25]. 

**Figure 2:**
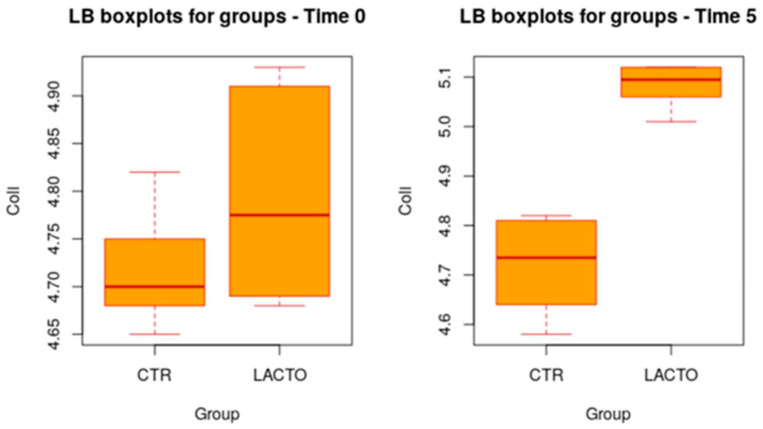
Box plot showing the effect of *Lactobacillus reuteri* NBF 2 DSM 32264 addition to diet on total *Lactobacilli* count (LB) during the overall period (*p* > 0.001; *t*-test). CTR, control group; LACTO, experimental group.

**Figure 3:**
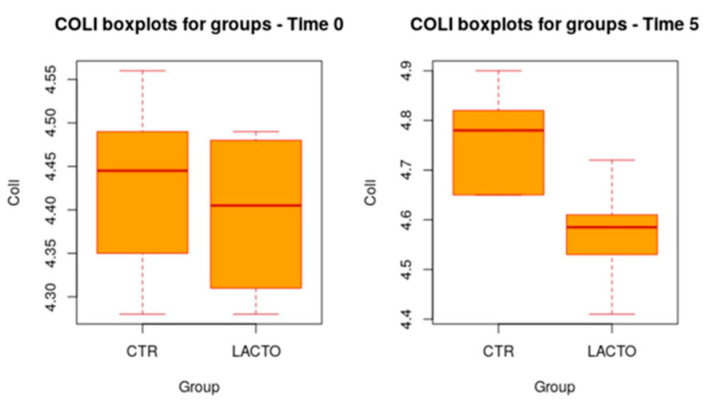
Box plot showing the effect of *Lactobacillus reuteri* NBF 2 DSM 32264 addition to diet on total coliform (Coli) during the overall period (*p* = 0.011; *t*-test). CTR, control group; LACTO, experimental group.

In addition, at the end of the study (T5), a more significant increase in *Lactobacilli* was reported in the experimental group (LACTO) than in the control group (*p* > 0.001), moving from a concentration of 4.75 ± 0.10 log CFU/g (*p* = 0.977) at the beginning of the experiment to a concentration of 5.04 ± 0.10 log CFU/g (*p* > 0.001) at the end of the trial (Table 6; Figure 2). Total coliforms show a very small increase (*p* = 0.011) (Table 7; Figure 3).

## 4. Discussion

The present study aims to evaluate the possible beneficial effects of the administration of a specific probiotic strain, *Lactobacillus reuteri* NBF 2 DSM 32264, on the fecal parameters of healthy adult Persian cats. This is the first study conducted on a specific breed of cat, the Persian one, and, at the same time, the first study that examines the effect of *L. reuteri* on cats; in fact, in the literature to date, there are no studies that analyzed the effects of *L. reuteri* administration on cats, especially on Persian cats. This aspect could be very important as in dogs several studies have already reported breeds more predisposed to others to suffer from gastrointestinal disorders, while in cats, this knowledge is still very limited, and the administration of a stabilizer of intestinal microflora might help one species more than another to maintain a balanced intestinal ecosystem. Furthermore, the studies conducted so far evaluated the effects of *L. acidophilus* administration on the fecal quality of healthy adult cats [20] but as it is known, there is a strain specificity, and the effects of *L. reuteri* could be different from those previously seen for *L. acidophilus*. However, it must be remembered that, unlike dogs, cats are animals, due to their digestive physiology, that are classified as obligate carnivores because their anatomical, functional and biochemical characteristics make them particularly suitable for a diet rich in feeds of animal origin (rich in proteins, fats and minerals) but with little presence of vegetable feed, which would provide starch and fiber. Therefore, cats that take a ration consisting of feed of animal origin show a high digestive capacity with the natural consequence of emissions of little fecal material with poor water content and are characterized by a low fecal score (on the evaluation scale from 1 to 7). In the case of cats fed with dry (extruded) feeds, as in the specific case of this test, we find in the composition of the feed a greater share of feeds of plant origin (starch in particular), which, however, following the heat treatment of the extrusion, undergoes a complete gelatinization with consequent improvement of digestibility. Dietary modulation is strictly connected to the quality and quantity of the fecal material [26], as the bacteria present in the last intestinal tract can ferment undigested food residues, influencing not only the fecal quality but also the entire intestinal ecosystem [27]. Often, the feed alone is not able to improve the fecal quality of these animals, and the use of lactic bacteria seems to be able to modulate the intestinal microbiota of cats [28]. *L. reuteri* NBF 2 DSM 32264, analyzed in this study, leads to a visible improvement in fecal parameters, showing the aforementioned consequences on the quality of the fecal material. *L. reuteri* NBF 2 DSM 32264 is not only able to modify the quality of the stool (consistency, color and compactness) but also to modulate the composition of the intestinal microbiota by influencing the number and species of microorganism present, as demonstrated by a previous study carried out on dogs [29]. In fact, *L. reuteri* NBF 2 DSM 32264 can reduce fecal moisture by improving the fecal score in the group of cats that took it, showing a fecal score very close to that considered ideal. The results obtained from the present study show how the intake of *L. reuteri* NBF 2 DSM 32264 promotes beneficial effects at the intestinal level of healthy Persian cats, making the feces more consistent and well-formed compared to those of the control group, thanks to the lower fecal humidity recorded, especially in the last two weeks of study (T4 and T5); obviously, at the same time, the fecal score also improved in the group of dogs treated with the probiotic, compared to the control one. At a microbiological level, *L. reuteri* NBF 2 DSM 32264 can increase the number of lactobacilli, especially in the last two weeks of the study, leading to a small decrease in total coliforms. The increase in lactobacilli is to be considered positive as they promote the integrity of the intestinal barrier, preventing the possible adhesion and proliferation of pathogenic bacteria [30]; furthermore, they can release antimicrobial substances and specific metabolites such as short-chain fatty acids, which are fundamental for intestinal well-being. The slight decrease in total coliforms recorded in both groups of cats could be correlated with the fact that the intestine naturally hosts this bacterial species, and it is normal to find an increase in them over time [31].

## 5. Conclusions

The data collected in this study report on the ability of the probiotic *L. reuteri* NBF 2 DSM 32264 to improve fecal quality parameters, such as FM and FS, in healthy adult Persian cats. The fecal score represents a very important parameter from a biological point of view closely related to the animal’s intestinal ecosystem status. At the end of the present study, there was a reduction of 0.7 points in the fecal score in the group of Persian cats that received the probiotic, accompanied by a reduction in fecal moisture values, leading to more compact and better-formed feces. In addition, from the fecal parameters analyzed, we can observe how the intake of *L. reuteri* NBF 2 DSM 32264 promotes better digestion and intestinal health related to an increase in lactobacilli and a decrease in total coliforms, highlighting that *L. reuteri* NBF 2 DSM 32264 is able to modulate the intestinal microflora composition increasing the number of bacterial species capable of maintaining the integrity of the intestinal barrier by limiting the proliferation of potentially pathogenic ones.

## Figures and Tables

**Table 1:** Age, sex and body weight of cats enrolled in the study.

Cats (CTR Group)	Age (Months)	Sex	Body Weight (Kg)
1	42.40	M	4.14
2	42.50	M	4.19
3	43.10	M	3.96
4	43.10	F	3.82
5	43.00	F	3.66
6	42.70	F	3.58
**Cats (LACTO Group)**	**Age (Months)**	**Sex**	**Body Weight**
7	43.60	M	4.21
8	43.70	F	3.56
9	43.40	F	3.68
10	43.50	F	3.58
11	42.90	F	3.49
12	43.30	F	3.62

**Table 2:** Analytical components of Farmina N&D prime feline chicken and pomegranate adult.

Parameter	Amount (%)
Crude protein	44.00
Crude fats	20.00
Humidity	8.00
Raw ash	8.50
Calcium	1.10
Phosphorus	0.90
Magnesium	0.08
Omega 6	3.30
Omega 3	0.90
DHA	0.50
EPA	0.30

**Table 3:** Effect of *Lactobacillus reuteri* NBF 2 DSM 32264 addition to diet on body weight (BW) (**A**) and body condition score (BCS) (**B**) of Persian cats.

(A) Effect of *Lactobacillus reuteri* on Body Weight (BW) (LS Mean ± SE).				
Time	CTR Group	LACTO Group	*p*-Value	Power
**Overall**	3.89 ± 0.11	3.85 ± 0.13	0.56	0.25
**T0**	3.89 ± 0.16	3.85 ± 0.18	0.99	0.25
**T1**	3.90 ± 0.16	3.85 ± 0.18	0.99	0.33
**T2**	3.89 ± 0.16	3.86 ± 0.18	0.99	0.19
**T3**	3.89 ± 0.16	3.85 ± 0.18	0.99	0.22
**T4**	3.88 ± 0.16	3.85 ± 0.18	0.99	0.23
**T5**	3.89 ± 0.16	3.85 ± 0.18	0.99	0.26
**(B) Effect of *Lactobacillus reuteri* on Body Condition Score (BCS) (LS Mean ± SE).**				
**Time**	CTR Group	LACTO Group	*p*-Value	Power
**Overall**	4.78 ± 0.20	4.68 ± 0.21	0.41	0.39
**T0**	4.67 ± 0.47	4.64 ± 0.50	1.00	0.08
**T1**	4.92 ± 0.47	4.72 ± 0.50	0.98	0.54
**T2**	4.75 ± 0.47	4.81 ± 0.50	0.99	0.13
**T3**	4.75 ± 0.47	4.56 ± 0.50	0.98	0.54
**T4**	4.83 ± 0.47	4.81 ± 0.50	1.00	0.08
**T5**	4.75 ± 0.47	4.56 ± 0.50	0.98	0.55

**Table 4:** Effect of *Lactobacillus reuteri* NBF 2 DSM 32264 addition to diet on fecal moisture in cats: results of mixed models showing least squares means ± SE in the CTR (control group) and LACTO (experimental group) cats, for the six individual sampling times and overall, throughout the study.

Time	CTR Group	LACTO Group	*p*-Value	Power
**Overall**	0.45 ± 0.01	0.42 ± 0.01	0.002	0.99
**T0**	0.45 ± 0.02	0.46 ± 0.02	1.00	0.27
**T1**	0.45 ± 0.01	0.45 ± 0.01	1.00	0.11
**T2**	0.45 ± 0.01	0.44 ± 0.01	0.875	0.84
**T3**	0.45 ± 0.01	0.41 ± 0.01	0.057	0.99
**T4**	0.45 ± 0.01	0.39 ± 0.01	0.001	1.00
**T5**	0.44 ± 0.02	0.38 ± 0.02	0.001	1.00

**Table 5:** Effect of *Lactobacillus reuteri* NBF 2 DSM 32264 addition to diet on the fecal score of Persian adult healthy cats: results of mixed models showing least squares means ± SE in CTR (control group) and LACTO (experimental group) cats, for the six individual sampling times and overall, throughout the study.

Time	CTR Group	LACTO Group	*p*-Value	Power
**Overall**	3.47 ± 0.15	3.17 ± 0.17	0.0068	0.99
**T0**	3.42 ± 0.42	3.53 ± 0.43	1.00	0.3
**T1**	3.50 ± 0.42	3.45 ± 0.43	1.00	0.23
**T2**	3.42 ± 0.42	3.20 ± 0.43	0.941	0.72
**T3**	3.42 ± 0.42	3.03 ± 0.43	0.465	0.98
**T4**	3.58 ± 0.42	2.95 ± 0.43	0.067	0.99
**T5**	3.50 ± 0.42	2.86 ± 0.43	0.067	1.00

**Table 6:** Effect of *Lactobacillus reuteri* NBF 2 DSM 32264 addition to diet, expressed as log CFU/g, on the total amount of *Lactobacilli* present in the intestinal microflora of Persian adult healthy cats: results of mixed models showing least squares means ± SE in CTR (control group) and LACTO (experimental group) cats, for the four individual sampling times and overall, throughout the study.

Time	CTR Group	LACTO Group	*p*-Value	Power
**Overall**	4.71 ± 0.05	4.91 ± 0.05	>0.001	1.00
**T0**	4.72 ± 0.10	4.75 ± 0.10	0.977	0.43
**T1**	4.72 ± 0.10	4.83 ± 0.10	0.241	0.99
**T3**	4.70 ± 0.10	5.01 ± 0.10	>0.001	1.00
**T5**	4.72 ± 0.10	5.04 ± 0.10	>0.001	1.00

**Table 7:** Effect of *Lactobacillus reuteri* NBF 2 DSM 32264 addition to diet, expressed as log CFU/g, on the amount of *E. coli* present in the intestinal microflora of Persian adult healthy cats: results of mixed models showing least squares means ± SE in CTR (control group) and LACTO (experimental group) cats, for the four individual sampling times and overall, throughout the study.

Time	CTR Group	LACTO Group	*p*-Value	Power
**Overall**	4.64 ± 0.05	4.53 ± 0.05	0.011	0.99
**T0**	4.43 ± 0.15	4.37 ± 0.15	0.981	0.41
**T1**	4.64 ± 0.15	4.69 ± 0.15	0.992	0.33
**T3**	4.72 ± 0.15	4.51 ± 0.15	0.106	0.99
**T5**	4.76 ± 0.15	4.55 ± 0.15	0.095	0.99

## Data Availability

The data are contained within the Article.
